# Rapid Systematic Review-Informed Multidisciplinary Expert Consensus on the Management of HPV-Positive Women with Low-Grade Cervical Lesions and the Role of a *Coriolus versicolor*-Based Vaginal Treatment

**DOI:** 10.3390/medicina62071283

**Published:** 2026-07-03

**Authors:** Javier Cortés, Nadia Nassar, Maria del Rosario Blasco, Rosario Castaño, Javier de Santiago, Ana Rosa Jurado, Fernando Losa, Luis Serrano

**Affiliations:** 1 Gynecologic Oncology, Private Practice (Laboratorio Citología Dr. Cortés), 07004 Palma, Spain; 2 Pathology Unit of the Lower Genital Tract, Hospital Clínico Universitario Lozano Blesa, 50009 Zaragoza, Spain; 3 Unidad de Gestión Centro de Salud Nueva Andalucía, 04006 Almería, Spain; 4 Palacios de Salud de la Mujer Institute, 28009 Madrid, Spain; 5 MD Anderson Cancer Center, 28033 Madrid, Spain; 6 Triay Medical Centre, 29670 Marbella, Spain; 7 Clínica Sagrada Familia, 08022 Barcelona, Spain; 8 HM Gabinete Velázquez, 28001 Madrid, Spain

**Keywords:** cervical lesions, *Coriolus versicolor*, human papillomavirus, vaginal gel, expert consensus, clinical management, psychological intervention

## Abstract

*Background and Objectives*: Persistent human papillomavirus (HPV) infection is associated with the development of cervical intraepithelial neoplasia and cervical cancer. In women with low-grade cervical lesions, a conservative management approach is commonly recommended. However, a subset of patients may experience lesion progression, which can also be accompanied by psychological distress. Adjuvant therapies during the surveillance period have gained increasing clinical interest. This consensus aims to provide multidisciplinary, expert-based recommendations on the use of a *Coriolus versicolor*-based vaginal gel in HPV-positive women with low-grade cervical lesions. *Materials and Methods*: A rapid systematic review was conducted in accordance with PRISMA guidelines, and the level of evidence was assessed using SIGN criteria. Subsequently, a multidisciplinary panel of experts formulated and evaluated consensus statements and recommendations using the nominal group technique and a voting process. *Results*: All statements reached consensus, with agreement levels exceeding 80%. Experts concluded that available evidence reports potential benefits of a *Coriolus versicolor*-based vaginal gel in HPV-positive women with low-grade cervical lesions, since it has demonstrated increased lesion regression and viral clearance rates. These benefits may be more pronounced in specific subgroups, particularly women over 40 years of age. Limited studies also suggest a positive effect on vaginal microbiota, which may contribute to both vaginal health and HPV clearance, as well as a reduction in perceived stress. The experts emphasized the importance of patient education, shared decision-making, and addressing psychosocial aspects, including emotional well-being and sexual health. *Conclusions*: This consensus provides a multidisciplinary perspective on the management of HPV-positive women with low-grade cervical lesions, highlighting the adjuvant role of a *Coriolus versicolor*-based vaginal gel during surveillance. Although current evidence suggests potential clinical benefits, further high-quality, independent studies are needed to corroborate these findings.

## 1. Introduction

Human papillomavirus (HPV) infection is one of the most common sexually transmitted infections, and it is responsible for nearly all cases of cervical cancer (CC) [1]. While up to 80% of women may be infected with HPV at least once, many clear the infection spontaneously due to the host immune response. However, persistent infection with high-risk (HR-HPV) types, especially 16 and 18, is a critical factor in the development of pre-cancerous cervical lesions and, ultimately CC [2].

Prophylactic HPV vaccination is currently the primary strategy for preventing HPV-related diseases. Strong evidence supports its effectiveness in reducing HPV infection, cervical intraepithelial neoplasia (CIN), and the incidence of CC, especially when administered to pediatric patients before the onset of sexual activity. However, although some studies suggest a possible benefit in reducing recurrence after treatment of cervical lesions, the evidence supporting a therapeutic effect of HPV vaccination in women with established HPV infection remains limited and inconclusive [3,4,5].

Pre-cancerous cervical lesions are classified cytologically as low-grade or high-grade squamous intraepithelial lesions (LSIL and HSIL, respectively), which correspond approximately to histological grades of CIN1 and CIN2/3. About 10–20% of LSIL will progress to HSIL, with an average risk of progression to CC of 20–30%, within a period of up to 10 years [6]. Despite the identification of several risk factors impacting HPV cervical lesions progression (i.e., HPV genotype, age, microbiota, lifestyle), it is still unpredictable which patients will progress to CC [2,7]. In Spain, approximately 2000 new cases of CC are diagnosed annually [8], with excisional treatment normally being the final option.

According to the Spanish Association of Cervical Pathology and Colposcopy (AEPCC), and in line with other international guidelines (i.e., the American Society for Colposcopy and Cervical Pathology, ASCCP), the management of cervical lesions follows a risk-based approach in which HPV genotype is a key determinant [9,10]. Specifically, patients with atypical squamous cells of undetermined significance (ASC-US) or LSIL associated with HPV 16/18 infection have a higher immediate risk of underlying HSIL/CIN3+ (approximately 5–10%) and are therefore referred for colposcopy. In contrast, women with ASC-US or LSIL associated with other high-risk HPV types have a substantially lower immediate risk (approximately 0.15–5%) and are generally managed with active surveillance, including repeat HPV testing and cytology after 12 months [10].

From the patient’s perspective, the awareness of an HPV-positive (HPV+) infection without an active therapeutic intervention can lead to significant psychological distress, anxiety, feelings of shame and fear about cancer during the active surveillance period [11]. This emotional burden may negatively affect their quality of life (QoL), particularly in terms of psychological and social well-being, contagiousness and sexuality [12]. In addition, more screening programs worldwide have adopted HPV testing as a primary triage system [13,14], resulting in a progressive increase of HPV+ cases.

Given the emotional impact of an HPV+ result, the slow progression to CC, and the lack of established active treatments for HPV-positive women with low-grade cervical lesions, there is a need to optimize standard management strategies that address both clinical and psychological aspects of care.

In response to this unmet need, novel approaches have been developed for use during the active surveillance period [15,16,17]. These approaches aim to promote the regression of low-grade cervical lesions and reduce the risk of progression to high-grade disease. Among these innovative treatments, a *Coriolus versicolor*-based vaginal gel (Papilocare^®^) has been specifically designed and approved for the prevention and adjuvant treatment of HPV-induced lesions. This medical device creates a protective film on the cervical mucosa, creating a defensive barrier to prevent the risk of HPV integration. In this way, it disrupts HPV integration, prevents the formation of new lesions, and helps re-epithelialize pre-existing lesions. It also stimulates the rebalancing of the vaginal microbiota [18,19,20,21,22].

This consensus brought together a multidisciplinary panel of healthcare professionals (HCPs), including gynecologists, psychologists, sexologists and primary care physicians, to formulate evidence-informed statements based on both clinical judgement and available scientific literature. The panel addressed key aspects of HPV management, including patient education, psychological support, and the clinical role of adjuvant treatments. In particular, the experts assessed the efficacy, effectiveness and safety of a *Coriolus versicolor*-based vaginal gel, identified patient subgroups most likely to benefit, and evaluated its potential impact on clinical and psychological outcomes.

## 2. Materials and Methods

This document is the result of a multidisciplinary expert consensus developed using a nominal group technique (NGT). The expert panel comprised five gynecology specialists (one specialist in oncological gynecology, three gynecologist experts in cervical pathology and one gynecologist expert in natural ingredients), one primary care physician, one sexologist doctor, one midwife, one psychologist, and one expert patient. Two members of the expert panel were designated as coordinators and assumed leadership roles throughout the project. Participants came from different regions of Spain, and represented a variety of healthcare settings (i.e., primary care consultations, public and private hospitals, psychiatric units or nursing homes) providing a broad perspective on the management of HPV-positive women.

Consensus statements were based on clinical expertise and available literature evidence. A research protocol was designed to define project objectives and methodology, with input and validation from all panel members. Eleven clinical questions categorized into five topics were formulated using the PICO (Patient, Intervention, Comparison, Outcomes) or PICo (Population of Interest, Phenomenon of Interest, Context) formats [23] (Table 1, Supplementary Information Section S1). Firstly, the initial topics and questions were formulated and validated by the two coordinators, and afterwards by the rest of the members of the expert panel during a joint virtual meeting. Following agreement on the clinical questions, a rapid systematic review of the available literature was performed, and the quality of the identified evidence was critically assessed. The consensus statements were developed based on the synthesis of the collected data and the results of the assessment. Finally, a structured voting process using the NGT led to formal agreement on the final statements.

### 2.1. Rapid Systematic Literature Review

A rapid systematic review was conducted in September 2023 to support the development of the expert consensus, following the recommendations of the Preferred Reporting Items for Systematic Reviews and Meta-Analyses (PRISMA) [24]. To address specific questions, a search strategy was developed (Supplementary Information, Section S1). Briefly, searches were conducted in specialized clinical practice guideline databases such as Medline, National Guideline Clearinghouse and TRIP database. Subsequently, a systematic electronic search of MEDLINE and Epistemonikos was performed to detect systematic reviews. For primary studies, MEDLINE was utilized through the PubMed platform, employing MESH terms and Boolean operators. To complete the review, a grey literature search using Google search engine, Google Scholar, ResearchGate and Science.gov was carried out.

Data extraction and verification were carried out independently by two reviewers. For each question, titles and abstracts were initially screened for relevance. Selected references then underwent a full-text screening to confirm eligibility, requiring adherence to all inclusion criteria and no exclusion criteria, with discrepancies resolved by a third reviewer. The literature was synthesized through a narrative review to support the expert consensus evaluation, as no quantitative synthesis or meta-analysis were performed.

### 2.2. Assessment of Evidence Quality: Level of Evidence and Grade of Recommendation

The Scottish Intercollegiate Guidelines Network (SIGN) approach was used to establish the level of evidence (ranged from 1++ to 4, depending on the quality of the literature) and grade of recommendation (ranged from A—very strong to D—weak) (Supplementary Information, Section S3) [25]. The systematic review result was examined by all members of the expert panel prior to the final consensus meeting. Levels of evidence and degree of recommendation were assessed and validated. The expert panel generated initial evidence-based statements to answer the clinical questions formulated. In cases where the systematic review could not provide a definitive answer, expert opinion alone was considered.

### 2.3. Consensus: Nominal Group Technique

After formulating initial statements, a virtual consensus meeting was held with an external moderator, utilizing the NGT method to ensure fair and equal participation of all clinical experts [26,27].

During the meeting, the expert panel reviewed and discussed the proposed statements, and modifications were made in real-time to enhance clarity and facilitate agreement. Upon discrepancies, statements were further discussed until they were consensual or rejected, if no agreement existed. Anonymous voting was conducted on the resulting statements and conclusions, generating agreement percentages for each of them. Participants were requested to indicate their level of agreement on a 5-point Likert scale: 5 (strongly agree), 4 (agree), 3 (neither agree or disagree), 2 (disagree) and 1 (strongly disagree). For the domains in *Health information* and *Emotional management* all panel members indicated their (dis)agreement. For domains in *Efficacy*, *effectiveness and safety*, *Patient’s profile* and *Microbiota*, voting was limited to selected participants (all gynecologists, primary care physician, sexologist doctor and/or midwife).

### 2.4. Statistical Analysis

The project used a 5-point Likert scale to calculate the percentages of agreement and disagreement for each item. Consensus was considered as agreement greater than or equal to 80%, while 100% agreement was defined as unanimity. An agreement percentage between 66% and 79% was considered a discrepancy. Rejection was considered when the percentage of agreement was lower than 66%. The percentage of (dis)agreement for each statement was calculated based on the number of participants who responded to each item.

## 3. Results

### 3.1. Systematic Literature Review

**Figure 1:**
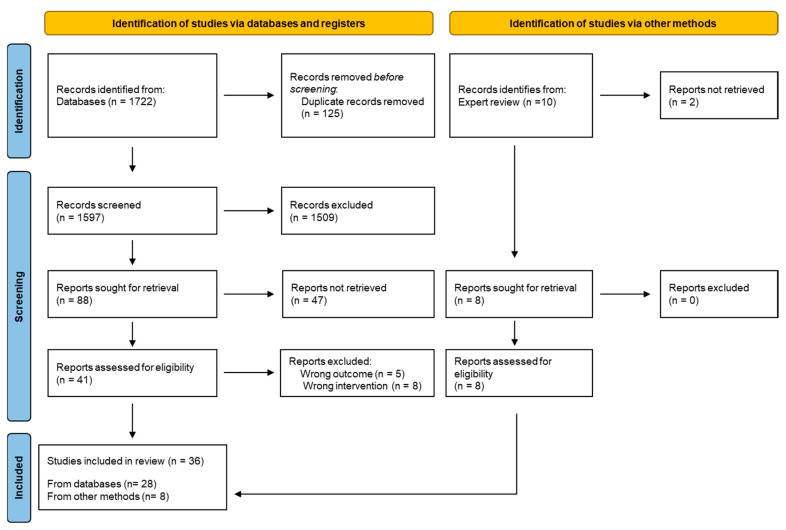
PRISMA flow diagram for systematic review.

A total of 1722 publications were identified from the selected databases. After removing duplicates (n = 125), 1597 publications were manually screened. Based on titles and abstracts, 1509 publications were excluded. Among the remaining 88 reports, 47 were excluded due to design issues, and afterwards 13 more were excluded for wrong outcomes or intervention, resulting in 28 reports. In parallel, 10 publications were included manually by the experts. After assessing these 10 studies, 2 were excluded due to design issues. Considering all, 36 records were included in the review (Supplementary Information, Section S2). Figure 1 shows the PRISMA flow diagram on rapid systematic review.

### 3.2. Evidence Quality Assessment

According to the expert panel and in accordance with the SIGN guidelines (Supplementary Information, Section S3), clinical questions about *Efficacy, effectiveness and safety* are supported by well-conducted, high- quality and low-biased literature (2++/2+). The participants agreed with the tolerability and positive benefits of the *Coriolus versicolor*-based vaginal gel for viral clearance and re-epithelization (grade of recommendation B-C scores). For the *Patient’s profile* statement, literature evidence was the best scored as 1−/B, revealing the high quality of the collected clinical data. On the other hand, *Microbiota* questions obtained a grade of recommendation of D (very weak), since the level of evidence was based on limited quality studies scored as 3. Regarding the *Health information* block, the rapid systematic review could not provide a definitive response, and results were based on expert opinion. Finally, *Emotional management* statements faced similar issues on limited systematic search. Thus, two clinical questions showed results based on expert opinion due to the lack of data, while one question was scored as moderate evidence with well-conducted studies (2+/C) (Table 2).

### 3.3. Consensus Process Results

From the formulated clinical questions, 10 statements were derived and categorized into five topics, as shown in Table 3, along with the percentage of agreement and the rationale.

All statements were accepted by consensus, with a degree of agreement greater than or equal to 80%. Two recommendations, included in the block *Emotional management*, obtained 89% of agreement while the remaining nine recommendations reported unanimity (100%). No statements were rejected.

## 4. Discussion

The World Health Organization (WHO) has already announced the commitment of the Spanish healthcare system to reduce CC, reaching an incidence of 5.3 cases per 100,000 women, close to the WHO’s goal of 4 cases per 100,000 women [28]. Scientific consensuses play a crucial role in the development of national and international health frameworks, as they support complex decision-making processes by revealing clinical evidence and expert opinion [29]. This is particularly relevant in secondary prevention, where early detection and appropriate management strategies can significantly impact patient outcomes.

In this context, the present consensus addressed an important gap in established clinical and psychological management of HPV-positive women with low-grade cervical lesions, as well as the potential benefits of adjuvant treatment with a *Coriolus versicolor*-based vaginal gel during the surveillance period. Although this consensus is aligned with Spanish clinical guidelines, these frameworks are consistent with major international guidelines, including those from ASCCP [9], the European Society of Gynaecologic Oncology (ESGO) [30], and the WHO [31]. All of them emphasize conservative management of low-grade cervical abnormalities and avoidance of overtreatment in transient HPV infections. Within this globally accepted paradigm, adjuvant strategies such as a *Coriolus versicolor*-based vaginal gel may represent a proactive option for selected patients.

### 4.1. Efficacy, Effectiveness and Safety

Low-grade cervical lesions are characterized by high spontaneous regression rates (approximately 60%), especially in young women under 30 years of age. However, regression rates decrease with age and in the presence of persistent high-risk HPV infection, particularly types 16 and 18 [32]. Therefore, although active surveillance management remains the standard management strategy, individualized risk stratification is essential for clinical decision-making [10].

The expert panel considers that a *Coriolus versicolor*-based vaginal gel may represent a well-tolerated adjunctive option in clinical practice for HPV-positive women with low-grade cervical lesions, with potential to promote HPV clearance and to improve cervical re-epithelization and vaginal health.

Evidence supporting these statements includes randomized and observational studies. A randomized control trial showed that treatment with *Coriolus versicolor*-based vaginal gel for 6 months statistically increases lesion regression (84.9% vs. 64.5%, *p* = 0.118) and improves viral clearance (59.6% vs. 41.9%, *p* = 0.076) compared to the control group, which followed a watchful waiting approach [18]. A retrospective observational study reported statistical conversion of HPV DNA testing to negative (67.0% vs. 37.2%, *p* < 0.0001) and regression in colposcopy (60.9% vs. 40.8%, *p* = 0.05) and cytology (70.8% vs. 34.8%, *p* < 0.0001) in the treatment group compared to control at 6 months [33]. Another observational study without a control group reported similar results in terms of HPV clearance (58.7% (95% CI: 51.7–65.8%)) and lesion regression (67.0% (95% CI: 60.4–73.7%)) at 6 months, although direct comparison cannot be made due to differences in the nature of the study and methodological design [21]. Importantly, these benefits appear to extend to women with high-risk HPV infection, since the studies reviewed reported similar significant improvements in lesion regression and increased clearance rates in this population. However, genotype-specific outcomes were not reported. Evidence from case reports also supports potential use of a *Coriolus versicolor*-based vaginal gel in specific situations such as pregnancy, the presence of HSIL [34], and condylomas [35], although these data remain limited.

In addition, treatment with this vaginal gel has demonstrated a favorable safety and tolerability profile. In a retrospective observational study involving 183 patients, no adverse events were reported [33]. Similarly, a prospective observational study including 201 patients in the safety population identified only three product-related adverse events (1.5%), all of which were mild to moderate in severity and mainly consisted of vulvovaginal itching or stinging [21]. Consistent with these findings, a randomized clinical trial reported seven possible or probable treatment-related adverse events, most of which (n = 6) were mild or moderate in severity [18]. Overall, the available data indicates that the *Coriolus versicolor*-based vaginal gel is well tolerated and that the incidence of treatment-related adverse effects is low.

### 4.2. Patient Profile

*Coriolus versicolor*-based vaginal gel treatment has been associated with favorable safety and effective clinical benefits in HPV-positive women aged from 30 to 65 years old [18,21,33]. Available evidence suggests that this approach may also be beneficial in the subpopulation of HPV-positive patients over 40 years old, where significant improvements in cytological and colposcopic findings (92.3% vs. 50%, *p* = 0.007) have been noted. In terms of HPV clearance, higher rates were reported in the treated group than in the untreated control group (61.5% vs. 50%, *p* = 0.725) [20]. Other studies support these findings, highlighting that a remarkable 74.0% (95% CI: 63.9–84.0%) of women over the age of 40 experience repairing of the lesions, and that 61.1% (95% CI: 49.9–72.4%) clear HPV after 6 months of treatment with the vaginal gel [21].

Older women are more susceptible to develop CC since spontaneous clearance of HPV infections reduces with age, most probably due to the decline of the immune system and the reactivation of latent infections [36,37]. Every 5 years, the probability of lesion regression is reduced by 21% [38,39]. Therefore, age is an important factor to consider for HPV persistence and progression to CC.

Based on evidence, the experts recommend the use of a *Coriolus versicolor*-based vaginal gel for a subpopulation of patients that can benefit most from the treatment: specifically, women over the age of 40 who are HPV-positive and have low-grade cervical lesions.

### 4.3. Microbiota

Some studies have provided insights into the relationship between vaginal microbiota and HPV infection, suggesting that HPV infection may alter the composition of the vaginal microbiota [40], and that certain bacterial populations may be associated with an increased likelihood of lesion progression [41]. The connection between microbiota composition and clinical outcomes has been increasingly investigated, leading to the notion that modulating microbiota, including through vaginal or oral interventions, may constitute a beneficial complementary approach in the management of HPV infection and its clinical consequences [16,42].

Regarding the effects of a *Coriolus versicolor*-based vaginal gel, an observational pilot study carried out in 21 HPV-positive women without lesions, reported positive trends in the vaginal microbiota following the use of the gel, meaning a significant increase in the *Lactobacillus* spp. populations and decrease in the pathogenic bacteria, such as *Gardnerella vaginalis* [19]. Although the evidence supporting the statement on vaginal microbiota was graded as weak (3/D) due to the observational nature and small sample size of the study, the expert panel acknowledges the potential positive effect of the *Coriolus versicolor*-based vaginal gel on vaginal microbiota modulation and its possible contribution to improved clinical outcomes.

### 4.4. Health Information and Emotional Management

To date, there is no available data defining what health information should be provided to HPV patients.

Based on clinical expertise, the expert panel underlines the need to inform HPV diagnosed women regarding (i) significance of the HPV+ result, (ii) healthcare program process, (iii) sexual transmission and partner’s implications, (iv) symptomatology, (v) colposcopy, biopsy, treatments and their effects, including consequences on fertility, (vi) importance of healthy habits, and/or (vii) HPV prophylactic vaccination.

HPV vaccination represents a crucial prophylactic strategy, although its therapeutic role is limited. Ongoing research is focused on the development of experimental therapeutic vaccines, although none has yet been approved for clinical use. Retrospective evidence suggests that vaccination in HPV-positive women may help prevent new infections and may reduce the risk of progression to CIN2+, particularly following excisional treatment. However, current prophylactic vaccines do not eliminate existing HPV infections or treat developed lesions. The literature indicates that both HPV status and age are important determinants of vaccine efficacy, with the highest effectiveness observed in younger HPV-naïve individuals, whereas reduced effectiveness in older or HPV-positive populations [3,4,5]. Given these heterogeneous findings, healthcare providers should counsel patients on the potential benefits and limitations of vaccination based on individual clinical characteristics.

The panel also highlights the importance of psychological support and shared decision-making. This fact is crucial to improve clinical outcomes, as several studies relate mental distress in HPV-positive women with a decrease in the immune response [43,44,45], leading to enhanced HPV persistence [46].

Currently, there are no validated guidelines that specifically define standardized psychological assessment strategies or counseling protocols that should be routinely offered to HPV-positive women. Despite these limitations, the expert panel proposed a potential “HPV Psychosocial Management Algorithm,” which includes the following recommendations:

1. Incorporation of validated instruments to assess psychological distress and quality-of-life during clinical consultations, such as the Perceived Stress Scale (PSS) or the HPV-Impact Profile (HIP) questionnaire [11].
2. Implementation of a two-tiered intervention strategy based on the patient’s level of psychological distress, including referral to healthcare professionals with expertise in psychological support when necessary.
3. Provide clear information on available treatment options, including their role (e.g., adjunctive therapy), route of administration (oral, vaginal, etc.), and potential implications, as part of a coping strategy to reduce anxiety associated with conservative management.
4. Encouraging the patient’s active participation and shared decision-making throughout the clinical management process.

In the PALOMA trial, the psychological impact of *Coriolus versicolor*-based vaginal gel was assessed using the PSS-14. The results showed that a greater proportion of patients in the treatment group reported improvement in perceived stress compared with untreated women [18]. Furthermore, this randomized study evaluated treatment adherence and patient satisfaction as patient-reported outcomes, showing that 94.3% of patients were compliant and 86.5% were satisfied with the adjutant therapy during the 6-month active follow-up period. Similarly, an observational study reported high levels of patient satisfaction (7.9/10) and compliance (94.2%) at 6 months [21].

Based on these findings, the expert panel recommends the use of *Coriolus versicolor*-based vaginal gel, as it seems to be well received by patients, with high satisfaction and adherence rates. In addition, the vaginal gel may help reduce perceived stress in women diagnosed with HPV by offering them a proactive option rather than limiting them to passive conservative management.

### 4.5. Future Perspectives

The statements evaluated in this consensus were based on clinical expert opinions and supported by evidence ranging from 1 to 3. Further high-quality and larger randomized clinical studies are required to confirm these findings, particularly in specific populations such as HR-HPV-positive women and older patients. These studies may consider direct comparisons between *Coriolus versicolor*-based vaginal gel and other strategies (i.e., comparators, placebo) to evaluate relative efficacy, effectiveness, safety, and long-term outcomes. This will guarantee the production of well-designed scientific publications with a low risk of bias. This consensus primarily analyzes outcomes in terms of lesion regression and HPV clearance; however, it also highlights the need for future studies to more thoroughly evaluate additional clinically relevant endpoints, such as genotype-specific HPV clearance, viral persistence, CIN2+ progression, infection recurrences, and histological outcomes. Assessment of these parameters would provide a more comprehensive view of clinical efficacy. In addition, further assessment is needed regarding their effects on vaginal health and the vaginal microbiota, as well as the benefits of patient education and individualized psychological support. The inclusion of patient-reported outcomes—such as quality of life, sexual well-being, and psychological distress—will be essential to strengthen the conclusions obtained in this consensus.

### 4.6. Strengths and Limitations

To our knowledge, this is the first consensus aiming to shed light on planning multi-disciplinary management of HPV-positive women with low-grade cervical lesions. This consensus highlights the unmet needs in the diagnostic and therapeutic management of HPV-positive women and underscores the importance of establishing evidence-based statements to design and optimize future strategies targeting clinical and emotional aspects. However, several limitations should be acknowledged. One limitation of this consensus is the heterogeneity of the studies and the level of evidence supporting the statements, resulting in a limited grade of recommendation in some cases, such as microbiota and emotional health. Furthermore, the experts were representative of clinical practice across Spain; therefore, the conclusions of this consensus may not be generalizable to countries with different clinical practices. Eventually, there is a lack of equal representation of different disciplines on the expert panel, which could bias recommendations toward specific areas of medical care. Some degree of conflict of interest cannot be excluded due to authors’ involvement in related publications. As mentioned, further randomized controlled clinical trials, systematic reviews or meta-analysis, including larger sample sizes and broader geographic representation, should be conducted to validate the findings of this consensus. Special attention should be paid to topics with lower scores, as emerging literature would allow the revision and updating of the recommendations once more robust evidence becomes available.

## 5. Conclusions

The multidisciplinary composition of the expert panel allows the integration of clinically relevant perspectives for the management of HPV-positive patients with low-grade cervical lesions.

This consensus provides an initial and valuable framework supporting the use of a *Coriolus versicolor*-based vaginal gel as a potential adjunctive therapeutic option during active surveillance, given its possible benefits in both clinical outcomes and psychological well-being. Despite the limitations associated with the rapid systematic review methodology and the scarcity of available evidence in certain areas, these recommendations represent an important step toward integrating clinical, microbiological, and psychosocial dimensions into HPV management. The consensus also underscores the need to foster collaboration among diverse medical specialties, the patient’s participation for decision-making, and the individualized assessment for psychological intervention. Finally, this consensus promotes ongoing high-quality research that contributes to a more robust understanding of the efficacy and safety of a *Coriolus versicolor*-based vaginal gel in HPV-positive patients.

## Figures and Tables

**Table 1:** List of clinical questions with PICO/PICo strategies applied for evidence synthesis.

**Topic 1: Efficacy and safety**		**PICO/PICo Strategy**
**Question 1**	In HPV+ women with low-grade lesions during the watchful waiting phase recommended by current clinical practice guidelines, is Papilocare^®^ an effective therapeutic tool compared to the standard watchful waiting approach?	**Patient:** HPV-positive woman with low-grade lesions **Intervention:** Administration of Papilocare^®^ **Comparison:** Standard watchful waiting approach **Outcomes:** HPV clearance, viral elimination, prevention of lesion progression
**Question 2**	Is Papilocare^®^ a well-tolerated therapeutic tool in HPV+ women with or without low-grade lesions?	**Patient:** HPV-positive woman with low-grade lesions **Intervention:** Administration of Papilocare^®^ **Comparison:** Standard watchful waiting approach **Outcomes:** Adverse events, patient satisfaction
**Question 3**	In HPV+ women, does Papilocare^®^ provide additional benefits to these patients?	**Patient:** HPV-positive woman (without lesions or with low-grade lesions) **Intervention:** Administration of Papilocare^®^ **Comparison:** Standard watchful waiting approach **Outcomes:** Additional benefits
**Topic 2: Patient’s profile**		
**Question 4**	Which HPV+ patient profiles can benefit most from the treatment with Papilocare^®^?	**Patient:** HPV-positive woman (without lesions and with low-grade lesions) **Intervention:** Administration of Papilocare^®^ **Comparison:** Standard watchful waiting approach **Outcomes:** Patient profile
**Topic 3: Microbiota**		
**Question 5**	Is there a direct relationship between vaginal microbiota and HPV persistency/clearance?	**Patient:** Woman **Intervention**: Healthy vaginal microbiota **Comparison:** — **Outcomes:** Local immunity and viral clearance Considerations: A healthy vaginal microbiota may promote lesion regression and/or viral clearance.
**Question 6**	In HPV+ women, does Papilocare^®^ treatment improve vaginal microbiota?	**Patient:** HPV-positive woman **Intervention:** Administration of Papilocare^®^ **Comparison:** Standard watchful waiting approach **Outcomes:** Improvement of the vaginal microbiota Considerations: A healthy vaginal microbiota may promote lesion regression and/or viral clearance.
**Topic 4: Health information**		
**Question 7**	Do you think that the fact that an HPV+ woman is informed of all available hygienic-therapeutic measures and participates in decision-making can modify the regression of the lesions and reduce the HPV persistence?	**Population of interest:** HPV-positive woman **Phenomenon of interest:** Information about available hygienic and therapeutic measures for decision-making **Context:** Promotion of lesion regression and reduction of HPV persistence
**Question 8**	Which medical information should a patient receive when reporting a positive HPV test and/or a low-grade lesion?	**Population of interest:** HPV-positive woman **Phenomenon of interest:** Key medical information for the patient **Context:** Following an HPV-positive diagnosis
**Topic 5: Emotional management**		
**Question 9**	In the clinical management of an HPV+ woman, is it relevant to address the emotional and psychosexual condition of the patients?	**Population of interest:** HPV-positive woman **Phenomenon of interest:** Impact on quality of life, sexual and reproductive health, and mood **Context:** Emotional status of an HPV-positive woman Considerations: It was decided to focus the question more on quality of life, sexual and reproductive health.
**Question 10**	In HPV+ patients, is there evidence that shows that reducing anxiety and stress can favor the regression of the pathology?	**Population of interest:** HPV-positive woman **Phenomenon of interest**: Anxiety and stress **Context:** Disease regression
**Question 11**	Is Papilocare^®^ a valid tool, which can improve the quality of life of an HPV+ woman?	**Population of interest:** HPV-positive woman **Phenomenon of interest:** Anxiety and stress **Context:** Quality of life

HPV: Human papillomavirus.

**Table 2:** Assessment of the quality of the scientific literature identified in the systematic review using SIGN criteria.

Clinical Questions	Level of Evidence	Grade of Recommendation
**Topic 1: Efficacy and safety**		
Question 1	2++	B
Question 2	2++	B
Question 3	2+	C
**Topic 2: Patient’s profile**		
Question 4	1−	B
**Topic 3: Microbiota**		
Question 6	3	D
**Topic 4: Health information**		
Question 7	Expert Opinion	
Question 8	Expert Opinion	
**Topic 5: Emotional management**		
Question 9	Expert Opinion	
Question 10	Expert Opinion	
Question 11	2+	C

**Table 3:** Consensus results: Percentage of agreement and rationale for final statements.

Statement	% Agreement	Rationale
**Topic 1: Efficacy and safety**		
1.1 Papilocare^®^ is an effective and well-tolerated tool for the treatment of women with HPV+ and low-grade lesions.	100%	Expert consensus based on randomized and observational studies.
1.2 Papilocare^®^ is an effective and well-tolerated tool for promoting HPV clearance in women with low-grade lesions.	100%	Expert consensus based on randomized and observational studies.
1.3 The use of Papilocare^®^ in HPV+ women provides significant improvements in cervical re-epithelization and positive benefits in vaginal health.	100%	Expert consensus based on randomized and pilot studies.
**Topic 2: Patient’s profile**		
2.1 The use of Papilocare^®^ is particularly recommended in HPV+ women over 40 years old with low-grade cervical lesions.	100%	Expert consensus based on a randomized clinical trial that included a subgroup analysis of participants >40 years.
**Topic 3: Microbiota**		
3.1 Papilocare^®^ has demonstrated improvements in women’s vaginal microbiota.	100%	Expert consensus based on clinical experience, given the limited evidence available from observational studies.
**Topic 4: Health information**		
4.1 To optimize the management and progression of their condition, a woman with HPV+ status should receive information on all available hygienic-therapeutic measures and actively participate in decision-making.	100%	Expert consensus based on clinical experience, due to the lack of literature.
4.2 The medical information that a woman diagnosed with HPV+ should receive includes: - The significance of an HPV+ result and its relation to precursor lesions and cervical cancer. - Continuity and process of healthcare. - Information about the sexual transmission of the virus and its implications for partners. - Presence or absence of symptoms explanation. - Detailed information about colposcopy, biopsy, treatments, and their effects (including consequences for fertility). - Advice on smoking cessation, healthy diet, and barrier contraceptive methods. - Information about HPV prophylactic vaccination.	100%	Expert consensus based on clinical experience, due to the lack of literature.
**Topic 5: Emotional management**		
5.1 It is recommended to address the emotional, psychosocial and sexual state in HPV+ patients with or without associated lesions.	89%	Expert opinion based on clinical experience, due to the lack of literature.
5.2 Individualized psychological intervention is recommended for HPV+ patients who require it.	100%	Expert opinion based on clinical experience, due to the lack of literature.
5.3 Papilocare^®^ is a useful tool for reducing perceived stress in HPV+ women.	89%	Expert opinion based on a randomized clinical trial.

## Data Availability

Data sharing is not applicable to this article as no datasets were generated or analyzed during the current study.

## Supplementary Materials

The following supporting information can be downloaded at: https://www.mdpi.com/article/10.3390/medicina62071283/s1, Table S1: List of studies included or excluded in review; Table S2: Levels of evidence according to SIGN guidelines.

## Abbreviations

(HR)-HPV	(High risk)-Human Papillomavirus
LSIL	Low-grade Squamous Intraepithelial Lesions
HSIL	High-grade Squamous Intraepithelial Lesions
PRISMA	Preferred Reporting Items for Systematic Reviews and Meta-Analyses
SIGN	Scottish Intercollegiate Guidelines Network
CC	Cervical Cancer
ASC-US	Atypical Squamous Cells of Undetermined Significance
QoL	Quality of Life
HCPs	HealthCare Professionals
NGT	Nominal Group Technique
WHO	World Health Organization
CIN	Cervical Intraepithelial Neoplasia
AEPCC	Asociación Española de Patología Cervical y Colposcopia
ASCCP	American Society for Colposcopy and Cervical Pathology
ESGO	European Society of Gynaecologic Oncology
PSS	Perceived Stress Scale
HIP	HPV-Impact Profile

## References

[1] Nelson C.W., Mirabello L. (2023). Human papillomavirus genomics: Understanding carcinogenicity. Tumour Virus Res..

[2] Burd E. (2003). Human papillomavirus and cervical cancer. Clin. Microbiol. Rev..

[3] Gardella B., Gritti A., Soleymaninejadian E., Pasquali M.F., Riemma G., La Verde M., Schettino M.T., Fortunato N., Torella M., Dominoni M. (2022). New Perspectives in Therapeutic Vaccines for HPV: A Critical Review. Medicina.

[4] Yousefi Z., Aria H., Ghaedrahmati F., Bakhtiari T., Azizi M., Bastan R., Hosseini R., Eskandari N. (2022). An Update on Human Papilloma Virus Vaccines: History, Types, Protection, and Efficacy. Front. Immunol..

[5] Arbyn M., Xu L., Simoens C., Martin-Hirsch P.P. (2018). Prophylactic vaccination against human papillomaviruses to prevent cervical cancer and its precursors. Cochrane Database Syst. Rev..

[6] Chen Y., Dong Z., Yuan L., Xu Y., Cao D., Xiong Z., Zhang Z., Wu D. (2024). A comparative study of treatment of cervical low-grade squamous intraepithelial lesions (LSIL). Photodiagnosis Photodyn. Ther..

[7] Bogani G., Ghelardi A., Sopracordevole F., Annoni M., Ciavattini A., Giannella L., De Vincenzo R., Cattani P., Barbero M., Vercellini P. (2023). Human papillomavirus (HPV) vaccination: A call for action in Italy. Int. J. Gynecol. Cancer..

[8] Asociación Española Contra el Cáncer AECC Observatorio. Dimensiones del Cáncer. https://observatorio.contraelcancer.es/explora/dimensiones-del-cancer..

[9] Perkins R.B., Guido R.S., Castle P.E., Chelmow D., Einstein M.H., Garcia F., Huh W.K., Kim J.J., Anna-Barbara M., Ritu N. (2020). 2019 ASCCP Risk-Based Management Consensus Guidelines for Abnormal Cervical Cancer Screening Tests and Cancer Precursors. J. Low. Genit. Tract Dis..

[10] Asociación Española de Patología Cervical y Colposcopia (AEPCC) Guía de Cribado del Cáncer de Cuello del Útero en España, 2025. https://www.aepcc.org/aepcc-guias/.

[11] McBride E., Tatar O., Rosberger Z., Rockliffe L., Marlow L.M., Moss-Morris R., Kaurb N., Wadec K., Waller J. (2021). Emotional response to testing positive for human papillomavirus at cervical cancer screening: A mixed method systematic review with meta-analysis. Health Psychol. Rev..

[12] Coronado P.J., González-Granados C., Ramírez-Mena M., Calvo J., Fasero M., Bellón M., García-Santos J.F., Rejas-Gutiérrez J. (2022). Development and psychometric properties of the human papillomavirus-quality of life (HPV-QoL) questionnaire to assess the impact of HPV on women health-related-quality-of-life. Arch. Gynecol. Obstet..

[13] Gilham C., Sargent A., Kitchener H.C., Peto J. (2019). HPV testing compared with routine cytology in cervical screening: Long-term follow-up of ARTISTIC RCT. Health Technol. Assess..

[14] Koliopoulos G., Nyaga V.N., Santesso N., Bryant A., Martin-Hirsch P.P.L., Mustafa R.A., Schünemann H., Paraskevaidis E., Arbyn M. (2017). Cytology versus HPV testing for cervical cancer screening in the general population. Cochrane Database Syst. Rev..

[15] Major A.L., Mayboroda I., Riger A. (2023). Successful Preventive Treatment of Oncogenic Transforming HPV Infections in Low-Grade Cytology (ASC-US/LSIL) Patients with an Adsorptive and Antioxidant Vaginal Gel. J. Clin. Med..

[16] Lavitola G., Della Corte L., De Rosa N., Nappi C., Bifulco G. (2020). Effects on Vaginal Microbiota Restoration and Cervical Epithelialization in Positive HPV Patients Undergoing Vaginal Treatment with Carboxy-Methyl-Beta-Glucan. Biomed. Res. Int..

[17] Yang Y., Meng Y.L., Duan S.M., Zhan S.B., Guan R.L., Yue T.F., Kong L.-H., Zhou L., Deng L.-H., Huang C. (2019). REBACIN® as a noninvasive clinical intervention for high-risk human papillomavirus persistent infection. Int. J. Cancer..

[18] Serrano L., López A.C., González S.P., Palacios S., Dexeus D., Centeno-Mediavilla C., Pluvio C., Jesús D.L.F., Antonio L.J., Cristina V. (2021). Efficacy of a Coriolus versicolor-Based Vaginal Gel in Women with Human Papillomavirus-Dependent Cervical Lesions: The PALOMA Study. J. Low. Genit. Tract Dis..

[19] González S., Serrano L., Cortés J., Vezza T., Garrido-Mesa J., Algieri F., Morón R., Cabezas M.E.R., Gálvez J., Nogales A.R. (2022). Effect of a Coriolus versicolor-based vaginal gel on cervical epithelialization and vaginal microbiota in HPV-positive women: EPICERVIX pilot study. Acad. J. Health Sci..

[20] Gil-Antuñano S.P., Serrano Cogollor L., López Díaz A.C., González Rodríguez S.P., Dexeus Carter D., Centeno Mediavilla C., Martín P.C., de la Fuente Valero J., Fernández J.A.L., Barbat C.V. (2022). Efficacy of a Coriolus versicolor-Based Vaginal Gel in Human Papillomavirus-Positive Women Older Than 40 Years: A Sub-Analysis of PALOMA Study. J. Pers. Med..

[21] Cortés Bordoy J., de Santiago García J., Agenjo González M., Dexeus Carter D., Fiol Ruiz G., García Ferreiro C., Rodríguez S.P.G., Soteras M.G., Lamela E.M., Gil-Antuñano S.P. (2023). Effect of a Multi-Ingredient Coriolus-versicolor-Based Vaginal Gel in Women with HPV–Dependent Cervical Lesions: The Papilobs Real-Life Prospective Study. Cancers.

[22] Palacios S., Losa F., Dexeus D., Cortés J. (2017). Beneficial effects of a Coriolus versicolor-based vaginal gel on cervical epithelization, vaginal microbiota and vaginal health: A pilot study in asymptomatic women. BMC Womens Health.

[23] Richardson W.S., Wilson M.C., Nishikawa J., Hayward R.S. (1995). The well-built clinical question: A key to evidence-based decisions. ACP J. Club..

[24] Page M.J., McKenzie J.E., Bossuyt P.M., Boutron I., Hoffmann T.C., Mulrow C.D., Tetzlaff J.M., Akl E.A., Brennan S.E., Chou R. (2021). The PRISMA 2020 statement: An updated guideline for reporting systematic reviews. BMJ.

[25] Scottish Intercollegiate Guidelines Network (SIGN50) (2019). A Guideline Developer’s Handbook.

[26] Mullen R., Kydd A., Fleming A., McMillan L. (2021). A practical guide to the systematic application of nominal group technique. Nurse Res..

[27] McMillan S.S., Kelly F., Sav A., Kendall E., King M.A., Whitty J.A., Wheeler A.J. (2014). Using the Nominal Group Technique: How to analyse across multiple groups. Health Serv. Outcomes Res. Methodol..

[28] World Health Organization (WHO) (2024). Cervical Cancer Elimination Initiative. Commitments.

[29] Arakawa N., Bader L.R. (2022). Consensus development methods: Considerations for national and global frameworks and policy development. Res. Social. Adm. Pharm..

[30] McGee A.E., Alibegashvili T., Elfgren K., Frey B., Grigore M., Heinonen A., Jach R., Jariene K., Kesic V., Küppers V. (2023). European consensus statement on expert colposcopy. Eur. J. Obstet. Gynecol. Reprod. Biol..

[31] World Health Organization (2024). WHO Guideline for Screening and Treatment of Cervical Pre-Cancer Lesions for Cervical Cancer Prevention: Use of Dual-Stain Cytology to Triage Women After a Positive Test for Human Papillomavirus (HPV).

[32] Loopik D.L., Bentley H.A., Eijgenraam M.N., IntHout J., Bekkers R.L.M., Bentley J.R. (2021). The Natural History of Cervical Intraepithelial Neoplasia Grades 1, 2, and 3: A Systematic Review and Meta-analysis. J. Low. Genit. Tract Dis..

[33] Criscuolo A.A., Sesti F., Piccione E., Mancino P., Belloni E., Gullo C., Ciotti M. (2021). Therapeutic Efficacy of a Coriolus versicolor-Based Vaginal Gel in Women with Cervical Uterine High-Risk HPV Infection: A Retrospective Observational Study. Adv. Ther..

[34] De La Torriente-Benito C.B. (2023). Effect of a Coriolus Versicolor-based Vaginal Gel on High-grade Cervical Lesion During Pregnancy. Mater. Chem. Horizons..

[35] Blanco Z.E. (2022). Case Report: Vulvar Condylomas-New Local Synergistic Treatments. Obstet. Gynaecol. Cases Rev..

[36] Liu Z., Liang Q., Ren Y., Guo C., Ge X., Wang L., Cheng Q., Luo P., Zhang Y., Han X. (2023). Immunosenescence: Molecular mechanisms and diseases. Signal Transduct. Target. Ther..

[37] Doorbar J. (2023). The human Papillomavirus twilight zone–Latency, immune control and subclinical infection. Tumour Virus Res..

[38] Bekos C., Schwameis R., Heinze G., Gärner M., Grimm C., Joura E., Horvat R., Polterauer S., Polterauer M. (2018). Influence of age on histologic outcome of cervical intraepithelial neoplasia during observational management: Results from large cohort, systematic review, meta-analysis. Sci. Rep..

[39] Gravitt P.E., Winer R.L. (2017). Natural history of HPV infection across the lifespan: Role of viral latency. Viruses.

[40] Santella B., Schettino M.T., Franci G., De Franciscis P., Colacurci N., Schiattarella A., Galdiero M. (2022). Microbiota and HPV: The role of viral infection on vaginal microbiota. J. Med. Virol..

[41] Mitra A., MacIntyre D.A., Ntritsos G., Smith A., Tsilidis K.K., Marchesi J.R., Bennett P.R., Moscicki A.-B., Kyrgiou M. (2020). The vaginal microbiota associates with the regression of untreated cervical intraepithelial neoplasia 2 lesions. Nat. Commun..

[42] Palma E., Recine N., Domenici L., Giorgini M., Pierangeli A., Panici P.B. (2018). Long-term Lactobacillus rhamnosus BMX 54 application to restore a balanced vaginal ecosystem: A promising solution against HPV-infection. BMC Infect. Dis..

[43] Fang C.Y., Miller S.M., Bovbjerg D.H., Bergman C., Edelson M.I., Rosenblum N.G., Bove B.A., Godwin A.K., Campbell D.E., Douglas S.D. (2008). Perceived stress is associated with impaired t-cell response to HPV16 in women with cervical dysplasia. Ann. Behav. Med..

[44] Nelson E.L., Wenzel L.B., Osann K., Dogan-Ates A., Chantana N., Reina-Patton A., Laust A.K., Nishimoto K.P., Chicz-DeMet A., Pont N.D. (2008). Stress, immunity, and cervical cancer: Biobehavioral outcomes of a randomized clinical trail. Clin. Cancer Res..

[45] Lopez C.R., Antoni M.H., Seay J., Potter J., O’Sullivan M., Fletcher M.A., Pereira D., Whitehead N. (2013). Stress Management, Depression, and Immune Status in Lower-Income Racial/Ethnic Minority Women Co-infected with HIV and HPV. J. Appl. Biobehav. Res..

[46] Kuebler U., Fischer S., Mernone L., Breymann C., Abbruzzese E., Ehlert U. (2021). Is stress related to the presence and persistence of oncogenic human papillomavirus infection in young women?. BMC Cancer.

