# Supplementary material for: Rapid Systematic Review-Informed Multidisciplinary Expert Consensus on the Management of HPV-Positive Women with Low-Grade Cervical Lesions and the Role of a Coriolus versicolor-Based Vaginal Treatment
Source: Medicina (Kaunas). 2026 Jul 3;62(7):1283. doi: 10.3390/medicina62071283 (PMC13413809; doi:10.3390/medicina62071283)
Supplement: Supplementary file 1 [file medicina-62-01283-s001.zip › medicina-4314344-supplementary.pdf]

Supplementary Information

Rapid Systematic Review-Informed **Multidisciplinary Expert Consensus on the Management of HPV-Positive Women with Low-Grade Cervical Lesions and the Role of a *Coriolus versicolor*-based Vaginal Treatment**

Javier Cortés[1,*], Nadia Nassar[2,*], Maria del Rosario Blasco[3], Rosario Castaño[4], Javier de Santiago[5], Ana Rosa Jurado[6], Fernando Losa[7], Luis Serrano[8]

[1]Gynecologic Oncology, Private Practice (Laboratorio citología Dr. Cortés), 07004 Palma, Spain.
[2]Pathology Unit of the Lower Genital Tract. Hospital Clínico Universitario Lozano Blesa, Zaragoza. Spain.
[3]Unidad de Gestión Clinica Nueva Andalucia, 04006 Almeria, Spain.
[4]Palacios de Salud de la Mujer Institute, 28009 Madrid, Spain.
[5]MD Anderson Cancer Center, 28033 Madrid, Spain.
[6]Triay Medical Centre, 29670 San Pedro Alcántara, Marbella, Málaga, Spain.
[7]Clínica Sagrada Familia, Barcelona, 08022, Spain.
[8]HM Gabinete Velázquez, 28001 Madrid, Spain.
*These authors contributed equally to the manuscript.

Corresponding Author:

Nadia Nassar
Secretaría de Ginecología y Obstetricia. 4º Planta del Hospital Clínico Universitario Lozano Blesa., C. de San Juan Bosco, 15, 50009 Zaragoza
nadia_nassar@hotmail.com
Telephone: 976556400 ext. 164925, ext. 162214

Materials and methods

Section S1. Rapid Systematic Review Protocol

A rapid systematic review was conducted in September 2023 to support the development of the expert consensus. A rapid systematic review is a structured and transparent synthesis of the available scientific evidence, conducted using systematic review methods with methodological adaptations designed to accelerate the review process without compromising scientific rigor. Given the consensus-building nature of this review, some standard systematic review procedures were adapted. Specifically, no meta-analysis or quantitative synthesis was conducted, and the evidence was summarized using a narrative synthesis. Furthermore, no formal assessment of risk of bias was performed.

Based on the clinical questions **(Table 1)** considered relevant and prioritized by the coordinator and expert panel, a rapid systematic review process was conducted following the accepted PICO format (Patient, Intervention, Comparison, Outcome). Alternatively, in cases where qualitative research is included, the PICo format (Population of interest, Phenomenon of Interest, Context), mainly used in mixed-methods systematic reviews, was applied.

S1.1. Studies selection criteria

In order to respond to the list of clinical questions (**Table 1**) with the evidence and clinical experience of clinical experts, criteria were established to guide the selection of articles identified through the literature searches and to determine their eligibility for inclusion in the review.

S1.1.1. Types of studies

This review will consider quantitative, qualitative, and mixed-methods studies. Clinical practice guidelines (CPGs), consensus documents, systematic reviews, primary studies,

and other relevant sources from the grey literature will be included where their content contributes to addressing the research questions.

Note: Given the limited number of publications related to the medical device Papilocare®, it may be necessary to broaden the search strategy using additional related terms.

S1.1.2. Population

The review will consider adult female patients with HPV infection (HPV-positive) without lesions or with low-grade cervical lesions.

Pregnant women and patients with high-grade cervical lesions will be excluded.

S1.1.3. Types of Intervention and Comparators

Interventions

- Administration of Papilocare®.
- Assessment of the vaginal microbiota.

Comparators

- Standard watchful waiting management.

S1.1.4. Outcomes

Disease-related outcomes

- HPV clearance/elimination.
- Prevention of lesion progression.
- Lesion normalization/regression.

Treatment-related outcomes

- Occurrence of adverse events.
- Treatment adherence.
- Patient satisfaction with treatment.
- Additional therapeutic benefits.

Microbiota-related outcomes

- Improvement in vaginal microbiota.

S1.1.5. Phenomena of Interest

This review will consider studies investigating hygienic and therapeutic measures, medical information provided to patients, and quality-of-life-related aspects such as mood, stress, anxiety, and sexual and reproductive health.

S1.1.6. Context

Studies conducted in all countries and populations, irrespective of race or ethnicity, will be considered. Conventional healthcare settings, including primary care, hospital care, and community settings, will be included.

S1.2. Databases and Information Sources

To identify clinical practice guidelines, specialized databases such as the National Guideline Clearinghouse, the National Library of Guidelines, and the TRIP Database meta-search engine will be consulted.

To identify major systematic reviews, the databases Epistemonikos and MEDLINE via PubMed will be searched.

A free-text search strategy will be conducted in Epistemonikos, which identifies evidence relevant to healthcare decision-making through the regular screening of multiple databases, including:

- Cochrane Database of Systematic Reviews (CDSR)
- PubMed
- EMBASE
- Database of Abstracts of Reviews of Effects (DARE)

For the identification of primary studies, MEDLINE via PubMed will be searched using MeSH terms and Boolean operators. The CENTRAL database (Cochrane Central Register of Controlled Trials) may also be consulted for randomized controlled trials (RCTs). Additionally, Google search may be used.

Grey literature searches will include Google, Google Scholar, ResearchGate, Science.gov, and medical books and databases.

S1.3. Search Terms

Generic Keywords

After identifying the relevant information sources, specific filters and keywords were defined for the bibliographic search strategy. These keywords will be combined into search syntaxes to retrieve the relevant literature.

Target Population

The target population will be included in all search strategies. The following keywords and search terms will be used:

- **Humans:** “Humans”[MeSH] – “Adult”[MeSH].
- **Sex:** woman – Female -Feminine –"female"[MeSH Terms]) - "women"[MeSH Terms]) – “sex [MeSH Terms].
- **Pathology:** HPV+ - Human Papilomavirus Human papillomavirus 16* - Human papillomavirus 18* - "Human Papillomavirus Viruses"[Mesh] – “Papillomavirus Infections”[MeSH].
- **Lesions:** Cervical lesions - low-grade cervical lesions - low-grade cervical squamous intraepithelial lesion – cervical LSIL – LSIL HPV – intraepithelial neoplasia - “Squamous Intraepithelial Lesions of the Cervix / pathology”[MeSH] - “Squamous Intraepithelial Lesions of the Cervix / virology*”[MeSH] – “Uterine

Cervical Dysplasia / virology”[MeSH] - Uterine Cervical Neoplasms / virology”[MesH].

Interventions and Comparators

- **Treatment:**
  - Papilocare: Coriolus versicolor - Coriolus-versicolor-Based Vaginal Gel – vaginal gel - “Papillomaviridae / drug effects*”[MeSH] – “Papillomavirus Infections / drug therapy*”[MeSH] – “Polyporaceae”[MeSH] – “Vaginal Creams, Foams, and Jellies”[MeSH].
  - Microbiota: Vaginal microbiota – Vaginal HEalth – «Cervix Mucus / microbiology»[MeSH].
- **Comparator:**
  - Wait-and-see strategy: “Watchful Waiting”[MeSH].

Additional Terms for Specific Questions

This section will include specific keywords for the PICo questions.

- **Questions 7–8:** hygienical-(and therapeutical) measures – alternative measures – clinical decision-making – decision aid information – medical information – drug patient information. Cervical lesions regression – HPV clearance.
- **Questions 9–11:** QoL – Quality of Life – Sexual and reproductive Health – Mood – Anxiety and stress – Mental health.

S1.4. Filters

Time Filters

The usual validity period for scientific publications is generally considered to be five years. However, due to the limited available literature regarding *Coriolus versicolor-*

based vaginal gel, the search period will be extended to the previous ten years. The rapid systematic literature review was conducted in September 2023.

Language Filters

Studies published in English and Spanish will be included.

S1.5. Study Selection and Data Extraction

Following execution of the search strategy and removal of duplicates, the initial screening will be conducted based on article titles and abstracts. Subsequently, all articles that may meet the requirements will undergo a full-text review to confirm their relevance and eligibility based on the predefined inclusion and exclusion criteria. The final selection of studies will be based on this comprehensive full-text review.

S1.6. Risk of bias Assessment

The literature review was conducted as a rapid systematic review designed to support the consensus-building process. In this context, certain methodological adaptations were implemented, while preserving the methodological integrity and reliability of the evidence synthesis. In the context of a rapid systematic review, a risk of bias assessment was not performed as part of the methodological approach. All other stages of the review process, including study selection, data extraction, and evidence assessment, were conducted using predefined and transparent methodological procedures.

Section S2. Rapid Systematic Review Results - List of selected studies

The following list includes all documents identified and evaluated during the rapid systematic literature review process, including both the studies included in the narrative

synthesis of the evidence and those excluded, along with the reasons for exclusion. The synthesis of the evidence was conducted using a narrative approach, as no quantitative synthesis or meta-analysis was performed.

Table S1. List of studies included or excluded in review.

**Studies included in review**					
PMID	Title	Type of publication	Year	Journal	DOI
33746195	Efficacy of a Coriolus versicolor-Based Vaginal Gel in Women With Human Papillomavirus-Dependent Cervical Lesions: The PALOMA Study.	Randomized Control Trial (RCT)	2021	Journal of lower genital tract disease	DOI: 10.1097/LGT.0000000000000596
	Effect of a Multi-Ingredient Coriolus-versicolor-Based Vaginal Gel in Women with HPV--Dependent Cervical Lesions: The Papilobs Real-Life Prospective Study	Case report	2023	Cancers	DOI: 10.3390/cancers15153863
33367986	Therapeutic efficacy of a Coriolus versicolor-based vaginal gel in women with cervical uterine high-risk HPV Infection: A retrospective observational study	Case control	2021	Advances in Therapy	DOI: 10.1007/s12325-020-01594-6
	Effect of a Coriolus versicolor-based vaginal gel on cervical epithelialization and vaginal microbiota in HPV-positive women: EPICERVIX pilot study	Observational	2022	Academic Journal of Health Science	DOI: 10.3306/AJHS.2022.37.02.139
	Biochemical Mechanisms of Action of Natural Ingredients from the Vaginal Applicators for the Management of	Review	2021	Revista de Chimie	DOI:10.37358/RC.21.4.8466

	Preinvasive Cervical Lesions				
	Evaluation of the level of satisfaction of patients with the use of a newly marketed vaginal gel (Satisvag survey)	Qualitative study	2018	Gynecol Obstet	DOI:10.4172/2161-0932.1000467
28302110	Beneficial effects of a Coriolus versicolor-based vaginal gel on cervical epithelization, vaginal microbiota and vaginal health: a pilot study in asymptomatic women.	Pilot study	2017	BMC women's health	DOI: 10.1186/s12905-017-0374-2
35527233	Microbiota and HPV: The role of viral infection on vaginal microbiota.	Non-analytic	2022	Journal of medical virology	DOI: 10.1002/jmv.27837
36353544	Vaginal microbiota and HPV clearance: A longitudinal study.	Case control	2022	Frontiers in oncology	DOI: 10.3389/fonc.2022.955150
37076931	The interplay between human papillomavirus and vaginal microbiota in cervical cancer development.	Review	2023	Virology journal	DOI: 10.1186/s12985-023-02037-8
34980148	Dysbiosis of vaginal microbiota associated with persistent high-risk human papilloma virus infection.	Case report/Case series	2022	Journal of translational medicine	DOI: 10.1186/s12967-021-03201-w
36683675	Roles of vaginal flora in human papillomavirus infection, virus persistence and clearance.	Case report/Case series	2022	Frontiers in cellular and infection microbiology	DOI: 10.3389/fcimb.2022.1036869
32214382	Cervicovaginal microbiome and natural history of HPV in a longitudinal study.	Case control	2020	PLoS pathogens	DOI: 10.1371/journal.ppat.1008376
35980030	Assessing the Cervicovaginal Microbiota in the Context of hrHPV Infections: Temporal Dynamics and Therapeutic Strategies.	Review	2022	mBio	DOI: 10.1128/mbio.01619-22
30463989	Evaluation of the Associations Between Cervical	Case report/Case series	2019	Cancer prevention research	DOI: 10.1158/1940-6207.CAPR-18-0233

	Microbiota and HPV Infection, Clearance, and Persistence in Cytologically Normal Women.				
28302110	Beneficial effects of a Coriolus versicolor-based vaginal gel on cervical epithelization, vaginal microbiota and vaginal health: a pilot study in asymptomatic women.	Pilot study	2017	BMC women's health	DOI: 10.1186/s12905-017-0374-2
36095006	Reproductive health needs of Human papillomavirus (HPV) positive women: A systematic review.	Systematic review	2022	PloS one	DOI: 10.1371/journal.pone.0266819
33323451	Information needs among women taking part in primary HPV screening in England: a content analysis.	Qualitative study	2020	BMJ open	DOI: 10.1136/bmjopen-2020-044630
32665351	Women's experiences of the renewed National Cervical Screening Program in Australia 12 months following implementation: a qualitative study.	Qualitative study	2020	BMJ open	DOI: 10.1136/bmjopen-2020-039041
34779742	"I'm neither here, which would be bad, nor there, which would be good": the information needs of HPV+ women. A qualitative study based on in-depth interviews and counselling sessions in Jujuy, Argentina.	Qualitative study	2021	Sexual and reproductive health matters	DOI: 10.1080/26410397.2021.1991101
29969339	Psychosocial impact of human papillomavirus on women's sexual dissatisfaction and quality of life.	Qualitative study	2019	Journal of psychosomatic obstetrics and gynaecology	DOI: 10.1080/0167482X.2018.1470164
24671715	Impact of genital warts on emotional and sexual well-being differs by gender.	Qualitative study	2014	International journal of STD & AIDS	DOI: 10.1177/0956462414526706
26782607	Sexual Activity, Psychosexual Distress, and Fear	Qualitative study	2016	The journal of sexual medicine	DOI: 10.1016/j.jsxm.2015.12.012

	of Progression in Women With Human Papillomavirus-Related Premalignant Genital Lesions.				
35128756	Psychosocial impact of testing human papillomavirus positive in Australia's human papillomavirus-based cervical screening program: A cross-sectional survey.	Qualitative study	2022	Psycho-oncology	DOI: 10.1002/pon.5897
32594298	Psychological distress in cervical cancer screening: results from a German online survey.	Qualitative study	2020	Archives of gynecology and obstetrics	DOI: 10.1007/s00404-020-05661-9
24690225	'I don't care whether it's HPV or ABC, I just want to know if I have cancer.' Factors influencing women's emotional responses to undergoing human papillomavirus testing in routine management in cervical screening: a qualitative study.	Qualitative study	2014	BJOG : an international journal of obstetrics and gynaecology	DOI: 10.1111/1471-0528.12741
34766557	An online survey on emotions, impact on everyday life, and educational needs of women with HPV positivity or abnormal Pap smear result.	Qualitative study	2021	Medicine	DOI: 10.1097/MD.0000000000027177
25048000	Human papillomavirus-related psychosocial impact of patients with genital warts in China: a hospital-based cross-sectional study.	Qualitative study	2014	BMC public health	DOI: 10.1186/1471-2458-14-739
36294699	Efficacy of a Coriolusversicolor-Based Vaginal Gel in Human Papillomavirus-Positive Women Older Than 40 Years: A Sub-	RCT	2022	J. Pers. Med	DOI: 10.3390/jpm12101559

	Analysis of PALOMA Study				
	Effect of a Coriolus Versicolor-based Vaginal Gel on High-grade Cervical Lesion During Pregnancy	Case report	2022	Mater. Chem. Horizons	DOI: 10.22128/MCH.2022.600.1023
	Vulvar Condilomas - New Local Synergistic Treatments	Case report	2022	Obstet Gynecol Cases Rev	DOI: 10.23937/2377-9004/1410225
35228537	HPV infection alters vaginal microbiome through down-regulating host mucosal innate peptides used by Lactobacilli as amino acid sources	Non-analytic	2022	Nat Commun	DOI: 10.1038/s41467-022-28724-8
32332850	The vaginal microbiota associates with the regression of untreated cervical intraepithelial neoplasia 2 lesions	Prospective study	2020	Nat Commun	DOI: 10.1038/s41467-020-15856-y
34242500	The Influence of Psychological Stress on HPV Infection Manifestations and Carcinogenesis	Expert Opinion/Narrative Review	2021	Cell Physiol Biochem	DOI: 10.33594/000000395
36560927	A systematic assessment of stress insomnia as the high-risk factor for cervical cancer and interplay of cervicovaginal microbiome	Expert Opinion/Narrative Review	2022	Front Cell Infect Microbiol	DOI: 10.3389/fcimb.2022.1042663
31251820	Anxiety and distress following receipt of results from routine HPV primary testing in cervical screening: The psychological impact of primary screening (PIPS) study	Qualitative study	2020	Int J Cancer	DOI: 10.1002/ijc.32540
**Studies excluded in review – reason for exclusion**					
	The management of human papilloma virus infection: results of the paloma clinical trial and derived research projects	Communication	2022	Academic Journal of Health Sciences	Data included un the PALOMA 1 publication
36676192	Exploring the Bioactive Mycocompounds (Fungal Compounds) of	Review	2023	Life	Not relevant to clinical questions

	Selected Medicinal Mushrooms and Their Potentials against HPV Infection and Associated Cancer in Humans				
28932226	Immunomodulatory Properties of Coriolus versicolor: The Role of Polysaccharopeptide.	Review	2017	Frontiers in immunology	Not relevant to clinical questions
35944043	Vaginal microbiota and personal risk factors associated with HPV status conversion-A new approach to reduce the risk of cervical cancer?	Pilot study	2022	PloS one	Wrong intervention
37331103	Lactobacillus gasseri LGV03 isolated from the cervico-vagina of HPV-cleared women modulates epithelial innate immune responses and suppresses the growth of HPV-positive human cervical cancer cells.	Pilot study	2023	Translational oncology	Wrong intervention
35873158	Role of Immunity and Vaginal Microbiome in Clearance and Persistence of Human Papillomavirus Infection.	Non-analytic	2022	Frontiers in cellular and infection microbiology	Wrong outcome
34204294	Vaginal Lactobacilli and Vaginal Dysbiosis-Associated Bacteria Differently Affect Cervical Epithelial and Immune Homeostasis and Anti-Viral Defenses.	Non-analytic	2021	International journal of molecular sciences	Wrong intervention
33881145	Distinct Illness Representation Profiles Are Associated With Anxiety in Women Testing Positive for Human Papillomavirus.	Qualitative study	2022	Annals of behavioral medicine	Wrong intervention
31556840	How Do Women Interpret the NHS Information Leaflet about	Qualitative study	2019	Medical decision making	Not relevant to clinical questions

	Cervical Cancer Screening?				
30481121	Understanding Patients' Perspectives and Information Needs Following a Positive Home Human Papillomavirus Self-Sampling Kit Result.	Qualitative study	2019	Journal of women's health (2002)	Wrong intervention
35023843	A Counseling Mobile App to Reduce the Psychosocial Impact of Human Papillomavirus Testing: Formative Research Using a User-Centered Design Approach in a Low-Middle-Income Setting in Argentina.	Qualitative study	2022	JMIR formative research	Wrong intervention
29845650	Longitudinal psychosocial adjustment of women to human papillomavirus infection.	Qualitative study	2018	Journal of advanced nursing	Not relevant to clinical questions
24580554	The information management processes of women living with HPV.	Qualitative study	2014	Journal of health communication	Wrong intervention
36346742	Psychosexual Impact on a Sample of Hispanic Women With Human Papillomavirus.	Qualitative study	2023	Journal of lower genital tract disease	Wrong intervention
	Treatment of Erythroplasia and Cervical HPV in a Young Patient with a Coriolus versicolor Vaginal Gel	Case report	2023	2nd Edition Prize Dr. Eduardo Vilaplana<br><br>https://www.ginecarefmc.com/wp-content/uploads/2023/01/CC_Procare_english_2022.pdf	Wrong outcome
	Vaginal Gel with Coriolus versicolor in the Treatment of Vaginal Intraepithelial Neoplasia (VaIN) in a Menopausal Patient	Case report	2023	2nd Edition Prize Dr. Eduardo Vilaplana<br><br>https://www.ginecarefmc.com/wp-content/uploads/2023/01/CC_Procare_english_2022.pdf	Wrong outcome
	Resolution of Persistent HPV 53 Infection in an Immunocompromised Patient after Treatment with Papilocare	Case report	2023	2nd Edition Prize Dr. Eduardo Vilaplana https://www.ginecarefmc.com/wp-content/uploads/2023/01/CC_Procare_english_2022.pdf	Wrong outcome

	Treatment with Papilocare in a HIV-Positive Patient with Persistent HPV and Refractory Condylomas	Case report		2nd Edition Prize Dr. Eduardo Vilaplana https://www.ginecarefmc.com/wp-content/uploads/2023/01/CC_Procare_english_2022.pdf	Wrong outcome

Section S3. Evidence Quality Assessment (SIGN Guidelines)

Table S2. Levels of evidence according to SIGN guidelines.

Levels of evidence	
1++	High-quality meta-analyses, systematic reviews of RCTs or RCTs with a very low risk of bias
1+	Well-conducted meta-analyses, systematic reviews of RCTs or RCTs with a low risk of bias
1-	Meta-analyses, systematic reviews of RCTs or RCTs with a high risk of bias
2++	High-quality systematic reviews of case–control or cohort studies. High quality case–control or cohort studies with a very low risk of confounding, bias or chance and a high probability that the relationship is causal
2+	Well-conducted case–control or cohort studies with a low risk of confounding, bias or chance and a moderate probability that the relationship is causal
2-	Case–control or cohort studies with a high risk of confounding, bias or chance and a significant risk that the relationship is not causal
3	Non-analytic studies, e.g. case reports, case series
4	Expert opinion
Grades of recommendation	
A	At least one meta-analysis, systematic review or RCT rated as 1++, and directly applicable to the target population; or,<br><br>A systematic review of RCTs or a body of evidence principally consisting of studies rated as 1+, directly applicable to the target population and demonstrating overall consistency of results
B	A body of evidence including studies rated as 2++, directly applicable to the target population and demonstrating overall consistency of results; or<br><br>Extrapolated evidence from studies rated as 1++ or 1+
C	A body of evidence including studies rated as 2+, directly applicable to the target population and demonstrating overall consistency of results; or<br><br>Extrapolated evidence from studies rated as 2++
D	Evidence level 3 or 4; or<br><br>Extrapolated evidence from studies rated as 2+
